# Standardizing thoracic segmental spinal anesthesia: an encouraging initiative that warrants methodological rigor. Comment on “A bundle for thoracic segmental spinal anesthesia: it is time to move forward!”

**DOI:** 10.1186/s44158-025-00267-6

**Published:** 2025-07-23

**Authors:** Carmine Pullano

**Affiliations:** Department of Anesthesia Villa Pia Clinic, Rome, Italy

To the Editor,

I read with great interest Vailati et al. recent editorial, which proposed a 7-item “MEL bundle” to guide the adoption of thoracic segmental spinal anesthesia (TSSA) [1].

The authors deserve credit for writing on a topic that is both timely and clinically relevant, especially given the growing interest in regional anesthetic approaches for frail and high-risk patients.

The structured approach of the bundle is a good way to standardize a technique that is still highly variable and underutilized. The emphasis on patient selection, ethical issues, and team-based planning is particularly beneficial.

Nevertheless, I would like to offer some points that may help the methodological foundation of such an attempt initiative stronger.

While the proposed bundle reflects clinical reasoning, it currently lacks supporting outcome data. The authors refer to “three years of experience” but do not provide data about safety, feasibility, or clinical outcomes. Even providing case series or observational studies based upon their own experience could make the impact stronger and inspiring.

Many aspects of the so-called ‘MEL bundle’—such as hemodynamic monitoring, informed consent, and sedation procedures—are already consistent with best practices in both regional and general anesthesia. These measures are endorsed by several scientific societies and are also recommended in Enhanced Recovery After Surgery (ERAS) protocols. For this reason, it is essential to clearly define what differentiates TSSA from other procedures by specifying which elements are critical to TSSA and identifying where standard protocols require adaptation.

The word “bundle” has methodological implications that need clarification. According to the Institute for Healthcare Improvement (IHI) [2], a clinical bundle is a small group of evidence-based practices (typically 3–5) that, when done together and consistently, contribute to improved outcomes. Each component must be supported by high-quality evidence, and compliance should be tested using an “all-or-nothing” method. The “MEL bundle” well described by the authors lacks formal validation, compliance metrics and proof of impact in TSSA scenario. Because of this, it may currently be more appropriately described as a structured checklist or conceptual framework. This difference is important when talking about TSSA, which remains contentious due to the hazards to the anatomy, the chance of spinal cord injury and high spinal block, and the use of off-label intrathecal adjuvants. In this case, promoting widespread use without strong evidence and consensus-based guidelines could unintentionally put patient safety or legal protection at risk.

While spinal anesthesia for laparoscopic surgeries is feasible and may provide potential advantages over general anesthesia, its application in laparoscopies has not been thoroughly investigated [3].

TSSA is an advanced technique and only adequate providers with adequate training could perform it. TSSA creates safety concerns—particularly regarding the risk of spinal cord injury during thoracic puncture, high spinal block and the off-label use of intrathecal adjuvants. These issues necessitate explicit medico-legal guidance and institutional protocols to ensure both patient safety and provider protection.

The editorial gives a clinical rationale, but it does not address common barriers to adoption, such as gaps in training, lack of ultrasound expertise, or surgical team hesitancy. Without addressing these, the call to 'move forward' risks sounding less like a push for progress and more like a polite suggestion to quietly abandon the technique.

In summary, I highly endorse efforts to develop and promote TSSA as a viable alternative approach, particularly for patients who are at high risk [4, 5]. I hope the authors will keep building their experience by releasing clinical trials or observational studies together with evidence-based guidelines to ensure safe and widespread adoption.

## Data Availability

No datasets were generated or analysed during the current study.
